# Datasets of traits of zodariid spiders (Araneae: Zodariidae)

**DOI:** 10.1038/s41597-024-03730-1

**Published:** 2024-08-10

**Authors:** Stano Pekár

**Affiliations:** Department of Botany and Zoology, Faculty of Science, Masaryk University, Kotlářská 2, 61137 Brno, Czech Republic

**Keywords:** Entomology, Behavioural ecology

## Abstract

Species traits are essential for inferences on ecology and the evolution of organisms. Spiders are the most abundant and diversified terrestrial predators, playing an important role in a range of ecosystem services. Here, I present datasetse on all traits of zodariid spiders, which are known to be free-living ground-dwellers occurring on all continents (except Antarctica) with the highest species diversity in Australia. I collated the data from published resources. The datasets includes nearly 100 000 trait records on all (90) genera and almost all species (1249) of the family. The majority of the 88 traits collected are morphometric, followed by those relating to ecology, reproduction, and physiology. Morphometric traits were available for the majority of species. Other trait classes were only available for some species. I provide a standardized classification of selected categorical traits (habitat, microhabitat, retreat type, circadian activity, prey, primary defensive, and predators). This is the first complete database of traits of a whole spider family, which is available through the World Spider Trait database.

## Background & Summary

Species traits are essential for a variety of ecological and evolutionary inferences^1,2^. With over 200 years of research on arthropods, a variety of traits have been recorded and published. These data are scattered among thousands of papers across the globe, published in a wide diversity of languages. Such trait data often remain undisclosed to current researchers, thus hampering progress in our research. It is, therefore, imperative to collate published traits and deposit them in centralised open-access databases, so that we can focus on recording traits that have not yet been measured.

Recently, the World Spider Trait (WST) database, designed to store trait data on spiders, has been developed^3^. This database is designed to accommodate a variety of traits measured on various taxonomic levels. It continues to grow thanks to the contribution of a number of people across the globe. Currently, it includes almost 500 000 records for almost 13 000 species.

With more than 52 000 species belonging to 135 extant families^4^, spiders represent the most diversified and abundant terrestrial group of predators^5^. They play an important role in a variety of ecosystems^6^. Many spider families are small, having less than 100 species, but several are quite species rich. Among them, zodariid spiders are the 12th richest family with almost 1300 species classified into 90 genera. Of these, 772 species are known from both sexes, 281 species only from the male sex, 217 species only from the female sex, and 18 species only from the juvenile stage^4^.

**Fig. 1:**
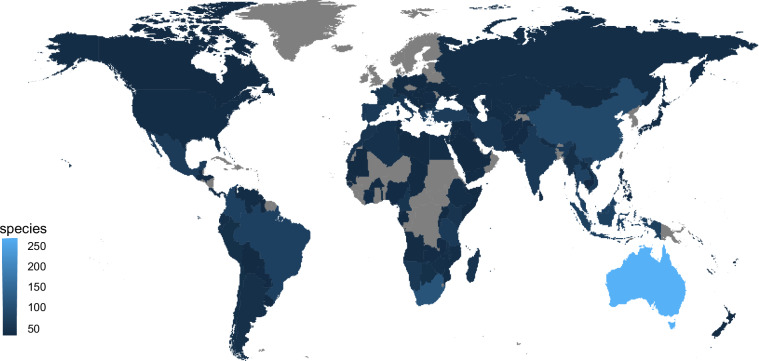
Map of species distribution of zodariid spiders. Based on World Spider Catalog^4^.

**Fig. 2:**
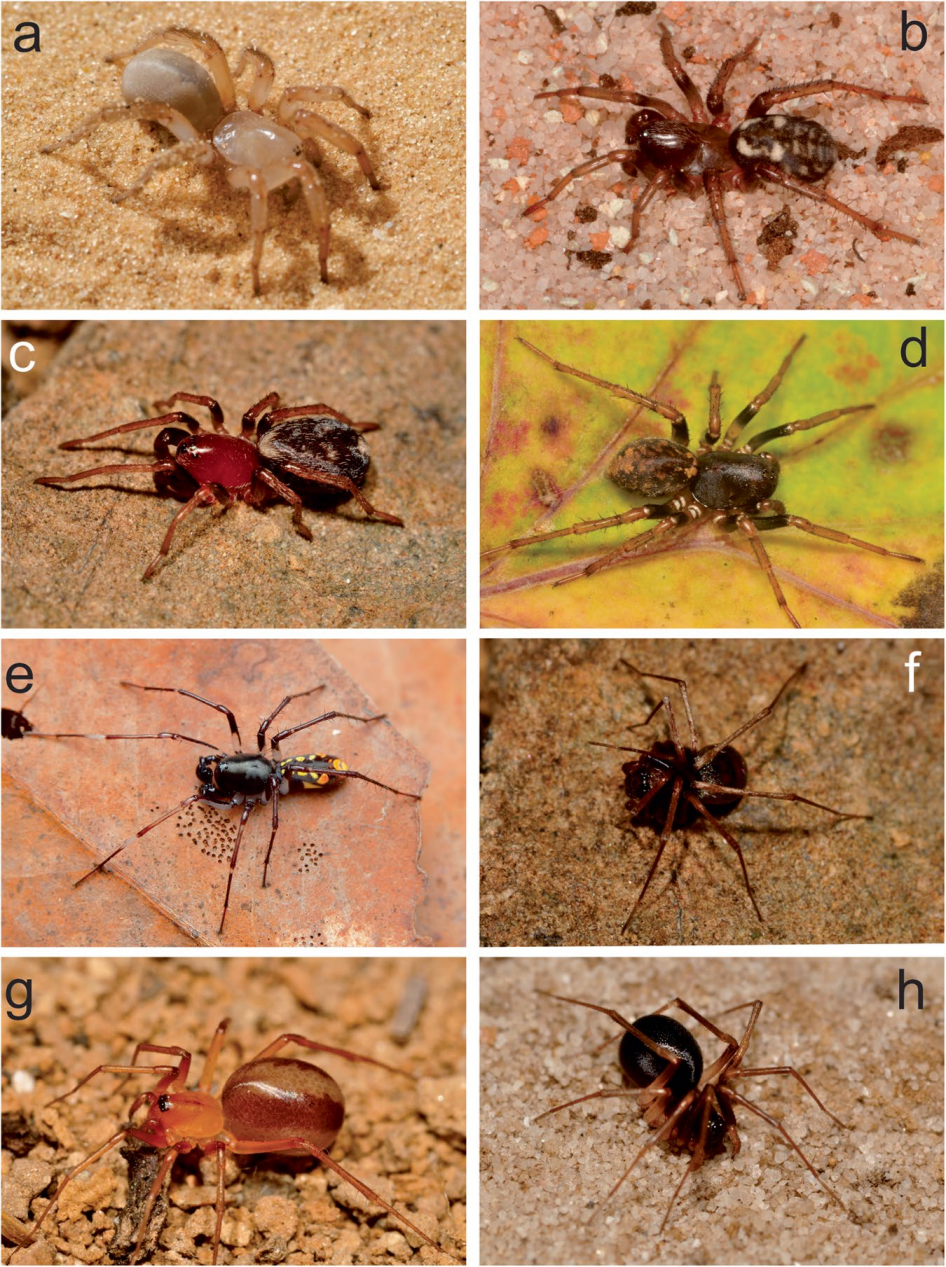
Habits of selected zodariid genera. (**a**) *Lachesana* sp., (**b**) *Cydrela* sp., (**c**) *Capheris* sp. (**d**) *Mallinella* sp., (**e**) *Subasteron* sp., (**f**) *Ranops* sp., (**g**) *Diores poweri*. (**h**) *Zodarion nitidum*. Photos: S. Pekár, O. Michálek.

Zodariids can be found on all continents (except for Antarctica), with the highest species richness in Australia, followed by South Africa and China (Fig. 1). Zodariids are small-sized, free-living, mostly ground-dwelling spiders^7^, most abundant in arid environments^8^. They have very variable phenotypes, particularly in morphology, with some species adapted to digging burrows while others are foliage hunters (Fig. 2).

The history of the taxonomic research of this family goes back to Walckenaer^9^, who described the first zodariid species (from Australia). About 70 years later, a solid family delimitation began to appear^10,11^; finally, Simon^12^ provided a complete description of the family. Another hundred years passed until Jocqué^13^ revised the whole family at the generic level.

Despite slow progress in their taxonomy, several other aspects have been intensively studied quite recently. As some zodariids are known to be ant-eating, the trophic ecology of several species has been studied over the last few decades^14–20^. This led to the discovery of morphological, behavioural, physiological and venomic adaptations related to trophic specialisation^21–23^.

Zodariid spiders have also been subject to toxinology research. The venom of one species has been investigated in detail and this led to the discovery of several new toxins^24–26^. In particular, the venom of ant-eating species holds the potential of including toxins with prey-specific effects^23,27^, which might be of particular interest for the development of bio-insecticides.

The aim of this study was to create an expert-curated global database of the traits of zodariid spiders. Such a collection of traits can be used to (1) study the (co)evolution of traits within this family, (2) compare traits with taxa from other families or taxon groups, and (3) answer specific questions concerning ecology and evolution. An additional aim was to standardize several traits that can be used in future studies. This is the first comprehensive database of traits of the species of one spider family.

## Methods

I collated traits from resources published until the end of 2023. I read through all taxonomic papers which were available through the World Spider Catalog^4^, then used Google Scholar and Zoological Records to search for additional non-taxonomic references using the keyword “Zodariidae”. Altogether, I found 519 references (including grey literature published in a variety of languages), from which morphological, physiological, ecological, and reproductive traits were extracted.

Detailed description of the traits is available on the WST webpage (https://spidertraits.sci.muni.cz/traits). Trait values were either single measurements, minimum and maximum values or means. Each trait record includes metadata – specifically, taxonomy, sex, developmental stage, methodology, country, and reference.

Altogether, I collected data for 88 traits belonging to eight categories (Table S1). For each trait I provide a basic summary, i.e. the number of records, the number of species, the number of genera for which the trait was reported, and the number of publications in which the particular category of traits was found (Table S1). For selected traits in most categories (which have been recorded for more species or have more levels), I estimated basic statistics presented mostly in the form of boxplots or pie charts. Traits which vary within a genus are presented at the species level, while those that are constant within the genus are presented at the genus level. In the case of size measures, I fitted a linear mixed-effect model (LME) of the ANCOVA type from the nlme package^28^ within the R environment^29^ in order to take into account the prosoma size, and used a nested taxonomic structure (species nested within genus) to estimate mean values. Then, I estimated an intraclass correlation coefficient (ICC) to estimate the within-genus covariation. I also estimated the Spearman correlation between eye sizes and prosoma length, or leg lengths and prosoma length, as the measurements had a skewed distribution.

Abbreviations used: AME – anterior median eyes, ALE – anterior lateral eyes, PME – posterior median eyes, PLE – posterior lateral eyes.

## Data Records

The data are stored in two main datasets^30,31^ deposited in the WST database^3^. The first dataset includes 42 323 records and the second includes 50 105 records. Another 7396 records of zodariid species has already been deposited in five datasets^32–36^ deposited in WST. All datasets can be downloaded from the WST database either in CSV or XLSX format. A detailed explanation of the variables in the dataset is given in the WST database (https://github.com/oookoook/spider-trait-database/blob/master/docs/template.md).

## Technical Validation

Original species names from the papers were checked against the World Spider Catalog^4^ when uploading the datasets into the WST. Each taxon was supplemented by its valid species name due to constant taxonomic changes. Whenever available, mean values of traits were accompanied by the sample size.

## Usage Notes

The selected traits can be effectively extracted/downloaded by means of functions from the arakno package^37^ within the R environment. Below, I provide an overview of the selected traits grouped by the trait classes.

### Morphology

The great majority of published traits are of a morphological character. Among 44 morphological traits, which appeared in 84% of publications, most data were available for body length (97% of species), cephalothorax length (96% of species), and cephalothorax width (96% of species). Other traits were available for approximately half of all zodariid species. For inferences on morphometric traits, it is essential to keep in mind that some measurements, such as eye diameter or leg length, are allometric to body size (e.g., prosoma length).

**Fig. 3:**
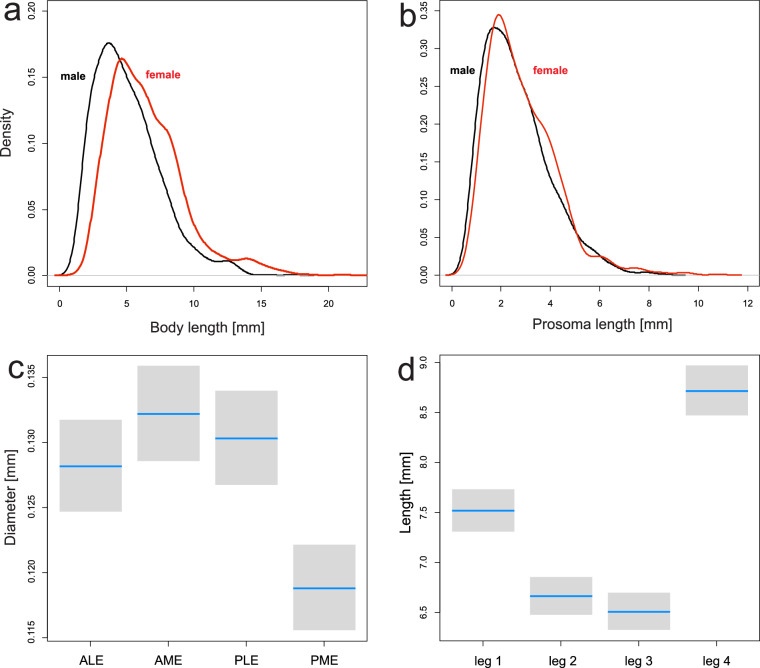
(**a**) Density diagram of total body length for males and females. (**b**) Density diagram of prosoma length for males and females. (**c**) Comparison of eye diameter of four eye types. (**d**) Comparison of leg lengths of four pairs. Blue lines are estimated means, grey boxes are 95% confidence intervals.

The average total body length of females is 6.2 mm (min-max: 0.75–22.1 mm) and that of males is 5.19 mm (1.0–19.5 mm) (Fig. 3a). The average prosoma length of females is 2.9 mm (0.6–10.7 mm) and that of males is 2.7 mm (0.6–9.1 mm) (Fig. 3b). There is sexual dimorphism in body/prosoma lengths, i.e. females are, on average, larger than males by 20%.

Zodariids possess 6 to 8 eyes with a variable eye arrangement which is constant within genera^13^. The diameter of eyes varies among genera. While in most genera the eyes are similarly sized, in e.g. *Diores*, *Ranops*, *Trygetus*, and *Zodarion*, AME are larger than other eyes. Overall, the diameter of eyes is strongly correlated to the prosoma length (Spearman correlation, *ρ* = 0.75, P < 0.0001, Fig. 3c). There is moderate covariation in eye diameter among species within a genus (ICC = 0.37), indicating that the diameters of particular eyes are weakly correlated. Across all species, the relative diameters of AME, ALE and PLE are not significantly different (LME, P > 0.05), but the relative diameter of PME is significantly smaller than that of other eyes (LME, P < 0.0001).

The length of legs varies but the pattern is rather constant among genera. The length of legs is strongly correlated to the prosoma length (Spearman correlation, *ρ* = 0.82, P < 0.0001, Fig. 3d). There is moderate covariation in leg length among species within genera (ICC = 0.41), revealing that the lengths of particular legs are rather weakly correlated. Across all species, leg pair I is significantly longer than leg pair II and III (LME, P = 0.0.03) but significantly shorter than leg pair IV (LME, P < 0.0001).

### Ecology

Among three ecological traits, which appeared in 25% of publications, most data were available for habitat and microhabitat type, a few for stratum (Table S1).

**Fig. 4:**
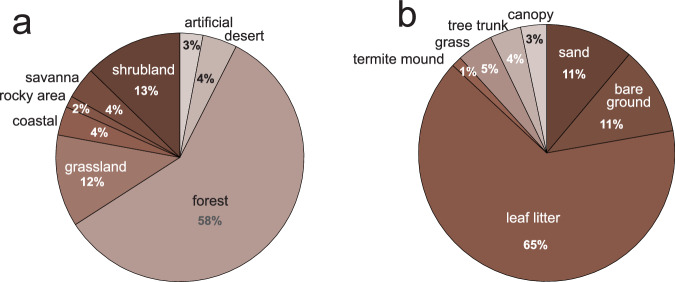
Relative frequency of habitats (**a**) and microhabitats (**b**) in which zodariid species were found.

Habitat data were available for 20% of species (76% of genera). The habitat types reported were re-classified according to the IUCN habitat classification scheme. Zodariid species can be found in a range of habitats, from very dry, such as deserts (e.g., *Cavasteron*, *Spinateron*, *Zodariellum*) to very humid, such as rainforests (e.g., *Euasteron*, *Euryeidon*, *Heliconilla*, *Mallinella*), and from pristine to artificial habitats, such as dumps (e.g., *Zodarion*) (Fig. 4a). A majority of species are found in forests, either in the tropical, subtropical, or temperate zone (e.g., *Ishania*, *Mallinella*, *Palindroma*, *Tenedos*). Shrubland species are found, for example, in the genera *Diores*, *Heradida*, *Masasteron*, and *Selamia*. Grassland species are found, for example, in the genera *Euasteron*, *Storena*, *Systenoplacis*, and *Zodarion*. The habitat type can even vary within a genus. Specifically, species of *Zodarion* can be found in all types of habitats reported for zodariids, except for rainforests.

With respect to microhabitat, data were available for 12% of species (66% of genera). The microhabitat types were classified into seven categories. The great majority of species are epigeic; only a very few are found in the canopy (e.g. *Mallinella*, *Suffasia*, *Tropasteron*). The majority of species of most genera are found in leaf litter (e.g., *Asceua*, *Heliconilla*, *Mallinella*, *Tenedos*), followed by the bare ground (e.g., *Palaestina*, *Trygetus*, *Parazodarion*, *Zodarion*) and sand (e.g., *Lachesana*, *Lutica*, *Pasammoduon*, *Psammorygma*) (Fig. 4b). The microhabitat type also varies within genera, with *Zodarion* species being found in all types of microhabitats, except for canopy.

### Defence

Among four defensive traits, which appeared in 10% of publications, most data were available for primary defence (2% of species), retreat type (2% of species), and predators (1% of species).

**Fig. 5:**
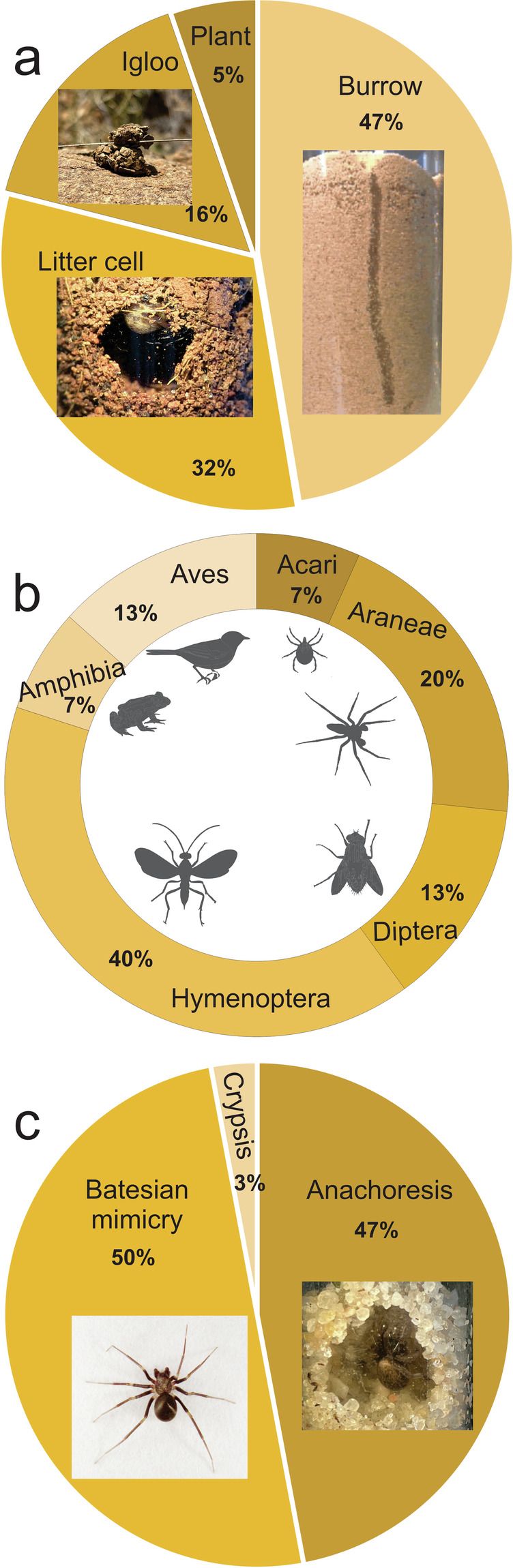
Relative frequencies of retreat types (**a**), predators (**b**), and primary defence strategies (**c**). Insets: a. Examples of retreats: igloo by *Diores*, litter cell of *Cydrela*, and burrow of *Lachesana*. c. Dorsal habitus of *Zodarion alacre* imitating ants, *Selamia* hiding in a litter cell.

Zodariid spiders hide in one of four retreat types (Fig. 5a), which are constant within genera. The great majority of genera hide in the ground, either in a cell built in litter or the upper soil layer (e.g., *Cydrela*, *Mallinella*, *Selamia*) or in deep burrows (e.g., *Antillorena*, *Cyrioctea*, *Lachesana*, *Lutica*). The cell or the burrow does not seem to be lined with silk as it collapses after prey capture, for example. Some genera (e.g., *Diores*, *Zodarion*) build igloo-shaped retreats which are stiff and compact, lined with dense silk threads, and attached, for example, to the lower sides of stones; a very small number of genera (e.g., *Cicynethus*) build retreats in plant stems.

Records of predators are rare but have been found for *Lutica*, *Systenoplacis*, and *Zodarion*. More than 50% of predators are parasitoids (hymenopteran or dipteran), followed by arachnids (spiders and mites) (Fig. 5b).

To defend themselves, zodariids use two major primary defensive strategies: Batesian mimicry is suspected to be used by diurnal species (e.g., *Acanthinozodium*, *Diores*, *Thaumastochilus*, *Zodarion*) (Fig. 5c), while anachoresis is used, for example, by *Cydrela*, *Lachesana*, *Lutica*, and *Selamia*.

### Predation

Among nine predation traits, which appeared in 13% of publications, most data were available for prey type (6% of species), prey diversity (3% of species), and circadian activity (2% of species). Only a few traits deserve detailed analysis as they show sufficient variation.

**Fig. 6:**
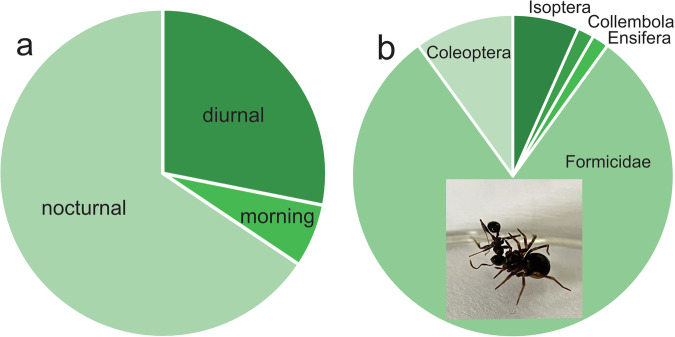
Relative frequencies of foraging activity modes (**a**) and prey orders (**b**). Inset: b. *Dusmadiores* feeding on an ant.

The circadian activity of zodariids may vary within the genus. Most species exhibit nocturnal activity, which has been reported for several genera (e.g., *Lachesana*, *Lutica*, *Mallinella*, *Storenomorpha*, *Zodarion*). Quite a few species exhibit diurnal activity (e.g. *Habronestes*, *Mallinus*, *Trygetus*, *Zodarion*), i.e. they forage in the morning and in the evening (Fig. 6a).

The prey diversity, estimated as the Shannon-Wiener index, revealed that the following genera are stenophagus (<1): *Acanthinozodium*, *Diores*, *Parazodarion*, *Zodarion*. A few other genera (e.g. *Lutica*, *Lachesana*, *Pax*, *Psammoduon*, *Selamia*) are euryphagous.

The great majority of species (e.g., *Acanthinzodium*, *Mallinus*, *Trygetus*, *Zodarion*) catch only ants though a few genera are also reported to catch beetles (e.g. *Lutica*, *Psammoduon*, *Selamia*), termites (e.g., *Diores*), and other arthropods (Fig. 6b).

### Reproduction

Among eight reproductive traits, which appeared in 3% of publications, most data were available for fecundity, mating duration, and egg size (all 1% of species).

**Fig. 7:**
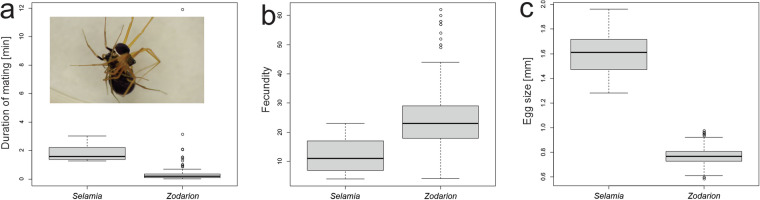
Boxplot of mating duration (**a**), number of eggs per sac (**b**), and egg size (**c**) of species of two genera, *Selamia* and *Zodarion*. Inset: (**a**) Mating in *Zodarion nitidum*.

Mating duration, number of eggs per sac and egg size were studied in only two genera (*Selamia* and *Zodarion*) (Fig. 7). Though *Selamia* mates for longer than *Zodarion*, it exhibits smaller clutch size but larger eggs.

### Physiology

Among 10 physiology traits, which appeared in 4% of publications, most data were available for sclerotization of the prosoma, venom gland size, toxin type, chromosome number, and sex chromosome system (all 1% of species).

The diploid chromosome number in seven *Zodarion* species varies between 21 and 42, with two types of sex chromosome determination systems (in males): X0 and X1X20.

**Fig. 8:**
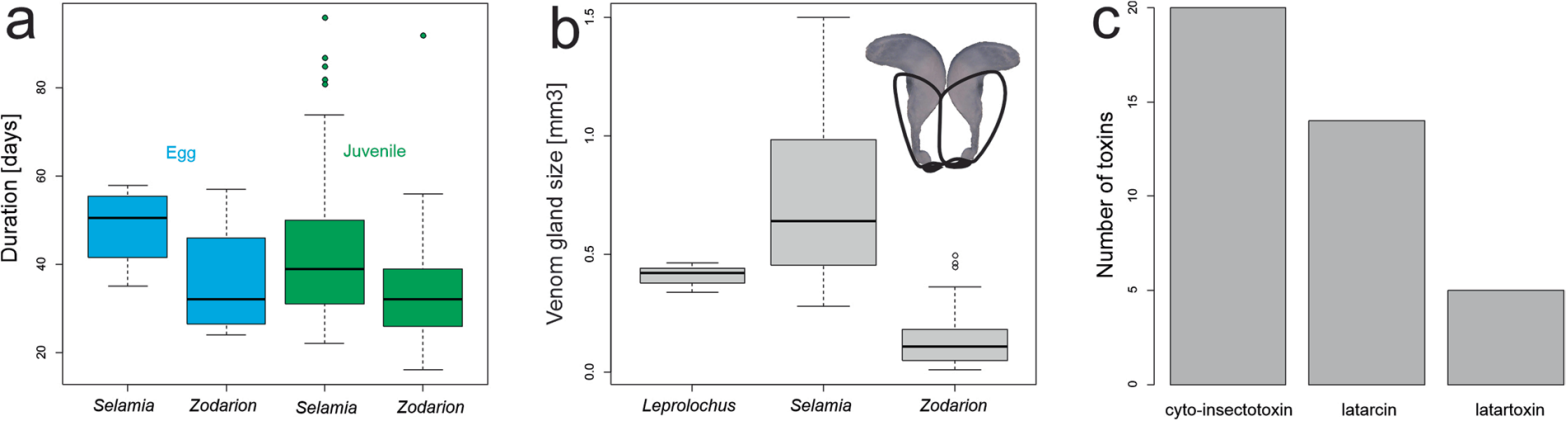
Boxplot of duration of one instar in two genera (**a,b**) venom gland size in three genera and barplot of number of toxins identified from *Lachesana tarabaevi* (**c**). Inset: (**a**) Venom glands of *Zodarion*.

The duration of incubation and the juvenile instar has been studied only in one species of *Selamia* and one species of *Zodarion*. Comparison shows that both incubation and instar duration are longer in *Selamia* than in *Zodarion*, most likely because the former is considerably bigger (Fig. 8a).

The venom gland has been studied in three genera (Fig. 8b). As its size is allometric to body size, it is larger for *Selamia* than for *Leprolochus* and *Zodarion* (Fig. 8b).

Venom toxins have been studied only in *Lachesana tarabaevi* and are of three types (Fig. 8c). The biggest category is cyto-insectotoxins, followed by latarcines, and latartoxins.

### Supplementary information

Table S1

## Data Availability

No specific code was developed in this work.
