# Supplementary material for: Datasets of traits of zodariid spiders (Araneae: Zodariidae)
Source: Sci Data. 2024 Aug 10;11:862. doi: 10.1038/s41597-024-03730-1 (PMC11316751; doi:10.1038/s41597-024-03730-1)
Supplement: Supplementary file 1 — Table S1 [file 41597_2024_3730_MOESM1_ESM.docx]

**Table S1.** Summary of collated traits grouped by trait category. For the definition of traits, see https://spidertraits.sci.muni.cz/traits.

| **Trait category/ Trait name (abbreviation)** | **No. of records** | **No. of genera** | **No. of species** | **No. of papers** |
| --- | --- | --- | --- | --- |
| **Biomechanics** |  |  |  | 2 |
| Acceleration (acce) | 30 | 2 | 2 |  |
| Angular velocity (anve) | 30 | 2 | 2 |  |
| Ballooning (balo) | 14 | 6 | 14 |  |
| Mobility (mobi) | 30 | 2 | 2 |  |
| Meander (mome) | 30 | 2 | 2 |  |
| Movement (move) | 30 | 2 | 2 |  |
| Running speed (rspd) | 60 | 2 | 2 |  |
| Total meander (tome) | 30 | 2 | 2 |  |
| Turn angle (tuan) | 30 | 2 | 2 |  |
| **Communication** |  |  |  | 1 |
| Sound source (sour) | 2 | 1 | 2 |  |
| **Defence** |  |  |  | 50 |
| Primary defence (prde) | 46 | 19 | 32 |  |
| Predator (pred) | 15 | 9 | 11 |  |
| Retreat (retr) | 27 | 18 | 25 |  |
| Secondary defence (sede) | 1 | 1 | 1 |  |
| **Ecology** |  |  |  | 130 |
| Habitat (habi) | 185 | 68 | 261 |  |
| Microhabitat (miha) | 156 | 59 | 150 |  |
| Stratum (strt) | 14 | 6 | 14 |  |
| **Morphology** |  |  |  | 433 |
| Abdomen length (able) | 934 | 45 | 547 |  |
| Abdomen width (abwi) | 731 | 39 | 419 |  |
| AME diameter (amed) | 1387 | 84 | 868 |  |
| ALE diameter (aled) | 1381 | 83 | 864 |  |
| Body colouration (cobo) | 1203 | 2 | 2 |  |
| Body mass (bodm) | 81 | 1 | 1 |  |
| Body length (bole) | 3579 | 90 | 1251 |  |
| Cephalothorax height (cehe) | 27 | 4 | 4 |  |
| Cephalothorax length (cele) | 3290 | 90 | 1240 |  |
| Cephalothorax width (cewe) | 2716 | 90 | 1234 |  |
| Clypeus height (clhi) | 915 | 62 | 540 |  |
| Chelicera length (chle) | 299 | 20 | 158 |  |
| Colouration of opisthosoma (coop) | 20905 | 2 | 6 |  |
| Colouration of prosoma (copr) | 20905 | 2 | 6 |  |
| Fang length (fale) | 27 | 6 | 6 |  |
| Sternum length (stle) | 724 | 49 | 430 |  |
| Sternum width (stwi) | 722 | 49 | 428 |  |
| Femur I length (fem1) | 1300 | 71 | 708 |  |
| Femur II length (fem2) | 1084 | 72 | 664 |  |
| Femur III length (fem3) | 1057 | 71 | 647 |  |
| Femur IV length (fem4) | 1124 | 71 | 691 |  |
| Legs I length (l1le) | 1134 | 73 | 681 |  |
| Legs II length (l2le) | 1125 | 72 | 671 |  |
| Legs III length (l3le) | 1125 | 72 | 670 |  |
| Legs IV length (l4le) | 1136 | 74 | 686 |  |
| Leg formula (lefo) | 5 | 1 | 3 |  |
| Patella I length (pat1) | 865 | 68 | 536 |  |
| Patella II length (pat2) | 791 | 68 | 485 |  |
| Patella III length (pat3) | 789 | 68 | 483 |  |
| Patella IV length (pat4) | 848 | 68 | 524 |  |
| PLE diameter (pled) | 1377 | 84 | 866 |  |
| PME diameter (pmed) | 1376 | 84 | 863 |  |
| Tibia I length (tib1) | 905 | 68 | 560 |  |
| Tibia II length (tib2) | 799 | 68 | 487 |  |
| Tibia III length (tib3) | 796 | 68 | 484 |  |
| Tibia IV length (tib4) | 860 | 68 | 528 |  |
| Metatarsus I length (met1) | 1111 | 71 | 688 |  |
| Metatarsus II length (met2) | 1043 | 71 | 639 |  |
| Metatarsus III length (met3) | 1040 | 71 | 637 |  |
| Metatarsus IV length (met4) | 1091 | 71 | 675 |  |
| Tarsus I length (tar1) | 1100 | 71 | 682 |  |
| Tarsus II length (tar2) | 1036 | 71 | 637 |  |
| Tarsus III length (tar3) | 1035 | 71 | 635 |  |
| Tarsus IV length (tar4) | 1085 | 71 | 672 |  |
| **Physiology** |  |  |  | 24 |
| Chromosome number (chrn) | 11 | 1 | 7 |  |
| Cuticular hydrocarbons (cuhy) | 28 | 1 | 1 |  |
| Instar duration (indu) | 117 | 2 | 6 |  |
| LD50 (ld50) | 3 | 2 | 1 |  |
| Sclerotisation (scle) | 14 | 6 | 14 |  |
| Sex chromosome system (chrs) | 9 | 1 | 7 |  |
| Sperm transfer form (stfo) | 1 | 1 | 1 |  |
| Toxin type (toxt) | 76 | 1 | 1 |  |
| Venom gland size (vgsi) | 65 | 3 | 10 |  |
| Venom profiling (vepr) | 9525 | 5 | 14 |  |
| **Predation** |  |  |  | 73 |
| Circadian activity (circ) | 236 | 13 | 30 |  |
| Prey consumption time (cons) | 20 | 1 | 2 |  |
| Kleptoparasitism (klep) | 5 | 1 | 2 |  |
| Prey paralysis latency (para) | 760 | 5 | 16 |  |
| Prey diversity (prdi) | 40 | 15 | 40 |  |
| Prey capture strategy (prec) | 6 | 4 | 5 |  |
| Prey order (preo) | 311 | 29 | 71 |  |
| Prey size (prsi) | 110 | 1 | 1 |  |
| Web-building (webb) | 14 | 6 | 14 |  |
| **Reproduction** |  |  |  | 13 |
| Courtship duration (codu) | 35 | 1 | 1 |  |
| Duration of mating (duma) | 109 | 2 | 8 |  |
| Number of eggs/sac (eggn) | 129 | 2 | 12 |  |
| Egg diameter (eggs) | 556 | 2 | 9 |  |
| Number of eggsacs (egsn) | 2 | 1 | 2 |  |
| Mating position (mapo) | 4 | 2 | 4 |  |
| Number of insertions (nuin) | 41 | 1 | 2 |  |
| Time to oviposition (ovip) | 4 | 1 | 2 |  |
| SUM | 99824 |  |  |  |
